# Study on Nuclear Magnetic Resonance Logging *T*_2_ Spectrum Shape Correction of Sandstone Reservoirs in Oil-Based Mud Wells

**DOI:** 10.3390/molecules26196082

**Published:** 2021-10-08

**Authors:** Jianmeng Sun, Jun Cai, Ping Feng, Fujing Sun, Jun Li, Jing Lu, Weichao Yan

**Affiliations:** 1 State Energy Center for Shale Oil Research and Development, Beijing 100083, China; sunjm@upc.edu.cn (J.S.); lijun67.syky@sinopec.com (J.L.); lujing.syky@sinopec.com (J.L.); 2 School of Geosciences, China University of Petroleum (East China), Qingdao 266580, China; b20010072@upc.edu.cn (F.S.); yanweichaoqz@163.com (W.Y.); 3 Shanghai Branch of CNOOC Ltd., Shanghai 200030, China; caijun@cnooc.com.cn

**Keywords:** NMR, oil-based mud, invasion correction, permeability

## Abstract

The oil-based mud filtrate will invade the formation under the overbalanced pressure during drilling operations. As a result, alterations will occur to the nuclear magnetic resonance (NMR) response characteristics of the original formation, causing the relaxation time of the NMR *T*_2_ spectrum of the free fluid part to move towards a slower relaxation time. Consequently, the subsequent interpretation and petrophysical evaluation will be heavily impacted. Therefore, the actual measured *T*_2_ spectrum needs to be corrected for invasion. For this reason, considering the low-porosity and low-permeability of sandstone gas formations in the East China Sea as the research object, a new method to correct the incorrect shape of the NMR logging *T*_2_ spectrum was proposed in three main steps. First, the differences in the morphology of the NMR logging *T*_2_ spectrum between oil-based mud wells and water-based mud wells in adjacent wells were analyzed based on the NMR relaxation mechanism. Second, rocks were divided into four categories according to the pore structure, and the NMR logging *T*_2_ spectrum was extracted using the multidimensional matrix method to establish the *T*_2_ spectrum of water-based mud wells and oil-based mud wells. Finally, the correctness of the method was verified by two *T*_2_ spectrum correction examples of oil-based mud wells in the study area. The results show that the corrected NMR *T*_2_ spectrum eliminates the influence of oil-based mud filtrate and improves the accuracy of NMR logging for calculating permeability.

## 1. Introduction

**Figure 1:**
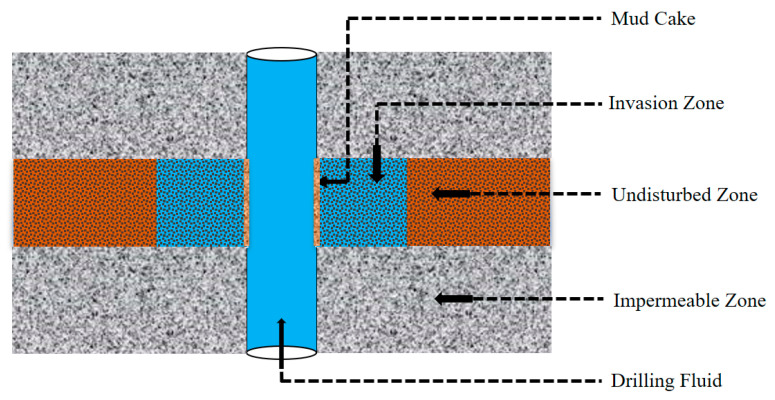
Schematic diagram of mud invasion process.

During drilling operations, drilling fluids are primarily used to ensure the smooth progress of drilling, and their importance is equivalent to the blood of the human body. The drilling fluid can carry out drilled cuttings, cool and lubricate the bits, balance and control the formation pressure, and maintain the stability of the wellbore [1]. To ensure the safety of drilling operation, the drilling fluid is usually circulated under a bottom hole pressure higher than the reservoir pressure, which is called overbalanced drilling [2]. Therefore, a fraction of the drilling fluid filtrate will penetrate the permeable formation under overbalanced pressure. Then, it will interact with the minerals and fluids in the formation rock, which will change the original petrophysical and geomechanical properties in the formation flushing zone [3]. The degree of invasion usually depends on two factors. The internal factors mainly include the quality control of reservoir formation, such as porosity, permeability, pore structure, original wettability of rock, and formation fluid properties. External factors are primarily controlled by drilling operation parameters, including drilling pressure, formation temperature, drilling time and drilling fluid properties [4]. On the walls of the wellbore, the solid particles in the mud are gradually deposited to form mud cakes. Because mud cakes are impermeable, when mud cakes are formed, they hinder the further invasion of mud filtrates [5]. As shown in Figure 1, this process will make the reservoir near the wellbore form several obvious annular areas: mud cake, invasion zone, undisturbed zone and impermeable zone.

Water-based mud (WBM) and oil-based mud (OBM) are considered the two types of drilling fluids that are most commonly used in the drilling process. Because shale oil reservoirs are rich in clay minerals and rock stability is poor, drilling with water-based mud will lead to water absorption and swelling in the rock, hydration expansion, borehole collapse, leakage, and so on [6,7]. Oil-based mud can effectively solve these problems, such as hydration expansion and wellbore instability of shale formations. Therefore, oil-based mud is most commonly used in the drilling process of shale oil reservoirs [8].

Nuclear magnetic resonance logging can directly measure the relaxation information of reservoir pore fluids and plays an extremely important role in the evaluation of petrophysical parameters, such as porosity, permeability, and saturation of various types of oil and gas reservoirs [9,10]. Based on the theory of NMR logging, the NMR transverse relaxation time (*T*_2_) is mainly affected by three relaxation mechanisms: bulk relaxation, surface relaxation, and diffusion relaxation, as shown in Equation (1):

(1) $$\frac{1}{T_{2}}=\frac{1}{T_{2B}}+\rho_{2}\frac{S}{V}+\frac{D{\left(\gamma GT_{E}\right)}^{2}}{12}$$

where $T_{2}$ is the transverse relaxation time, $T_{2B}$ is the bulk relaxation, $\rho_{2}$ is the surface relaxivity, *S/V* is the surface-to-volume ratio of the pore, D is the diffusion coefficient of the fluid, γ is the nuclear gyromagnetic ratio, G is the magnetic field gradient, and $T_{E}$ is the echo spacing of the measurement sequence.

However, due to the shallow detection depth of NMR logging, the *T*_2_ spectrum is easily affected by mud invasion. Especially in the environment of oil-based drilling fluids in shale oil reservoirs, the displacement of fluid in the flushed zone by oil-based mud filtrate may seriously affect the overall shape of the *T*_2_ spectrum and cannot accurately evaluate reservoir parameters [11].

**Figure 2:**
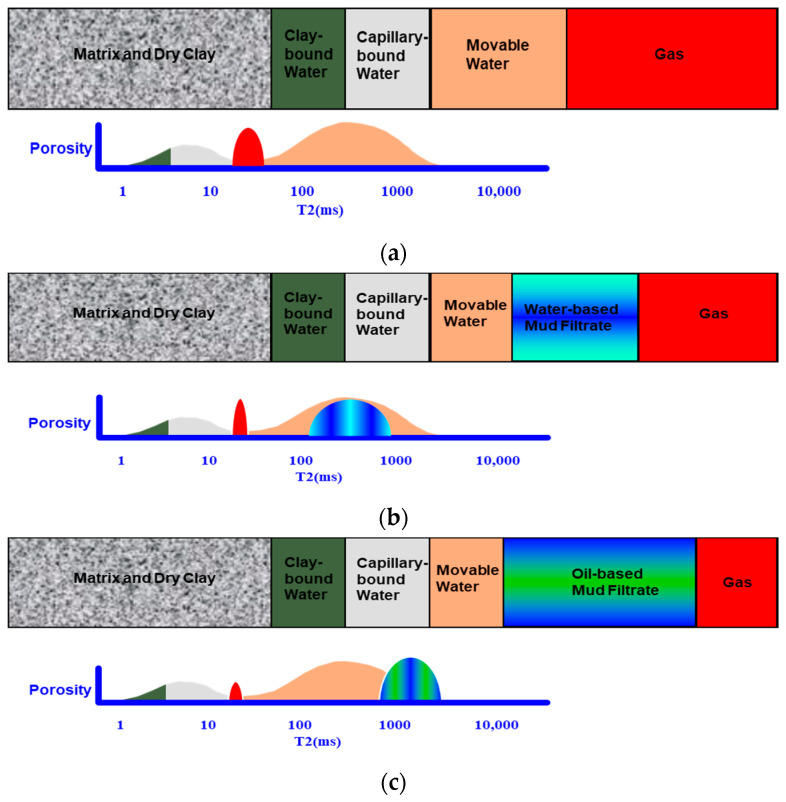
Gas reservoir volume model and theoretical NMR *T*_2_ spectrum diagram. (**a**) Volume model of gas-bearing reservoirs in undisturbed formations and theoretical distribution of the nuclear magnetic *T*_2_ spectrum. (**b**) Volume model of gas-bearing reservoirs in water-based mud wells and theoretical distribution of the nuclear magnetic *T*_2_ spectrum. (**c**) Volume model of gas-bearing reservoirs in oil-based mud wells and theoretical distribution of the nuclear magnetic *T*_2_ spectrum.

The study area belongs to a typical low porosity and low permeability sandstone gas reservoir with water content (the original water saturation range is 23–85%) and strong hydrophilic characteristics [12]. In this context, the rock volume model of the gas reservoir and the corresponding ideal NMR *T*_2_ spectrum were established, as shown in Figure 2. The model describes the ideal NMR *T*_2_ spectrum of gas-bearing reservoirs in undisturbed formations of water-based mud wells and oil-based mud wells.

1. The undisturbed formation does not contain mud filtrate, and the natural gas is a fluid of nonwetting phase in the formation pores of the study area. Its NMR signal only includes diffusion relaxation and free relaxation, which is not affected by surface relaxation. Due to the fast diffusion rate of natural gas, it has a short *T*_2_ time [13]. Figure 2a shows the rock volume model and ideal NMR *T*_2_ spectrum of the undisturbed formation, and the red part represents natural gas.
2. In the process of nuclear magnetic resonance logging, the response of the instrument will be affected by the mud filtrate in the invasion zone. Figure 2b shows the rock volume model and ideal nuclear magnetic resonance *T*_2_ spectrum in the water-based mud well: some natural gas and movable water in the formation invasion zone in the water-based mud well are displaced by the water-based mud filtrate, and the properties of the fluid are changed. The free gas product in the intrusion zone is less, and the signal of gas in the corresponding NMR *T*_2_ spectrum decreases. Because the wettability of the formation in the study area is strong water wet, the filtrate of water-based mud contacts the skeleton minerals on the pore surface, and its NMR response characteristics are affected by surface relaxation. Therefore, the *T*_2_ signals of the water-based mud filtrate and movable water overlap. For sandstone gas reservoirs, researchers usually use the *T*_2_ spectrum of nuclear magnetic resonance logging in water-based mud wells to calculate basic reservoir parameters.
3. Figure 2c shows the rock volume model and ideal nuclear magnetic resonance *T*_2_ spectrum in the oil-based mud well: in the oil-based mud well, in addition to some natural gas and movable water in the formation invasion zone being displaced by the oil-based mud filtrate, the oil-based mud filtrate will also be miscible with the residual natural gas in the invasion zone, resulting in a significant reduction in the natural gas signal collected by nuclear magnetic resonance logging. Because the filtrate of oil-based mud is in a nonwetting phase, the flow channel in the formation rock is the central area of the pore and does not contact the pore surface. Its NMR response is mainly characterized by volume relaxation. The viscosity of oil-based mud in the study area is 18 mPa.s, which is a low viscosity drilling fluid. Its body relaxation time is long, which significantly changes the *T*_2_ spectrum shape of the undisturbed formation. Therefore, if the existing reservoir parameter calculation methods are directly applied to the *T*_2_ spectrum of NMR logging in oil-based mud wells, it may lead to large calculation errors.

Considering the character analysis of nuclear magnetic resonance logging data in oil-based mud wells, many researchers have conducted related research [14,15,16,17]. Chen [14,15] believed that surfactants in oil-based drilling fluids would change the wettability of reservoir rocks, and thus using the default 33 ms as the *T*_2*cut-off*_ value for interpretation is no longer applicable; Marschall and Coats [16] believe that the macropores reflected by the *T*_2_ spectrum are greatly affected by the oil-based mud filtrate, while the small pores are not sensitive to the intrusion of the oil-based mud filtrate. However, the method of morphological correction of the *T*_2_ spectrum of nuclear magnetic resonance logging in oil-based mud wells is not well studied. Ighodalo [17] proposed a fluid substitution method to correct the nuclear magnetic *T*_2_ spectrum in oil-based mud wells, yet this method needs to accurately obtain the total water saturation in the invasion zone. Therefore, a method to quickly and accurately correct the shape of the NMR *T*_2_ spectrum after the invasion of oil-based mud filtrate in shale oil reservoirs is needed. However, due to the complex pore structure of shale oil reservoirs and the complex response mechanism of NMR logging, correction work is difficult. In this regard, this study proposed a new approach to correct the unreal NMR logging responses due to oil-based mud invasion in gas sandstone formations. By establishing the multivariate linear function relationship between the nuclear magnetic *T*_2_ spectrum of water-based mud wells and oil-based mud wells, the shape correction of the NMR logging *T*_2_ spectrum after the invasion of oil-based mud filtrates are realized, which improved the accuracy of NMR logging evaluation of reservoir parameters, and laid a foundation for the shape correction of the nuclear magnetic resonance logging *T*_2_ spectrum after the invasion of oil-based mud filtrates in shale oil reservoirs.

## 2. Results and Discussion

### 2.1. Comparison of NMR Logging Responses in Different Mud Environments

As shown in Figure 2, under the condition of drilling differential pressure, the original formation fluid was displaced by mud filtrate, and the properties of the formation fluid have changed. Moreover, the wettability of the formation determines the different distribution positions of oil-based mud filtrate and water-based mud filtrate in pores, resulting in the difference in the *T*_2_ spectrum morphology of NMR logging under different drilling fluid environments.

**Figure 3:**
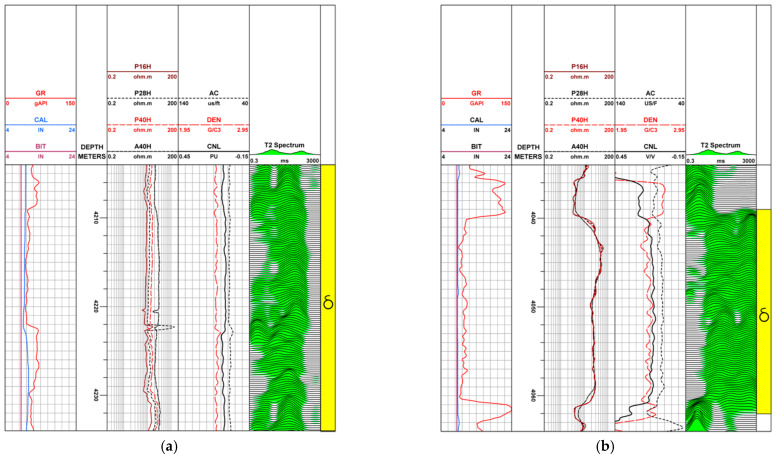
NMR logging response in different mud environments: (**a**) Water-based mud well A; (**b**) Oil-based mud well B.

In this study, the *T*_2_ spectrum characteristics of water-based mud well A and adjacent oil-based mud well B were compared and analyzed. Figure 3a is a comprehensive logging diagram of well A measured in a water-based mud environment. The sandstone interval is a gas reservoir, and its NMR *T*_2_ spectrum is distributed in a single peak. The *T*_2_ value of the main peak is less than 300 ms, and the maximum *T*_2_ value is less than 1000 ms. According to the volume model of gas-bearing reservoirs in water-based mud wells and the theoretical distribution of the nuclear magnetic *T*_2_ spectrum in Figure 2b, the distribution form of the nuclear magnetic *T*_2_ spectrum in water-based mud wells is basically not affected by the water-based mud filtrate and can be used for the calculation of reservoir petrophysical parameters and the evaluation of pore structure. A long relaxation time represents large pores, and a short relaxation time represents small pores [18].

Figure 3b is a comprehensive logging diagram of well B measured in the oil-based mud environment. The sandstone unit at interval 4039.0–4062.0 m is also a gas reservoir, but there is an obvious difference in NMR response characteristics from adjacent well A. Due to the influence of oil-based mud filtrate, there is a serious “tailing” phenomenon in the nuclear magnetic resonance *T*_2_ spectrum of this horizon. The *T*_2_ spectrum demonstrated a bimodal distribution. The *T*_2_ value of the second main peak is approximately 1000 ms, and the maximum *T*_2_ value is greater than 3000 ms. According to Figure 2c, the volume model of gas-bearing reservoirs in oil-based mud wells and the theoretical distribution of the nuclear magnetic *T*_2_ spectrum, the distribution form of the nuclear magnetic *T*_2_ spectrum in oil-based mud wells are obviously affected by the oil-based mud filtrate, so it is impossible to directly calculate reservoir petrophysical parameters.

### 2.2. Classification of Pore Structure Types of Reservoir Rocks

For the low-porosity and low-permeability reservoirs in the East China Sea, there is significant heterogeneity in the pore structure and lithology, and rocks with different lithologies and pore structures are affected by the invasion of oil-based mud filtrate. Therefore, the shape correction of the nuclear magnetic *T*_2_ spectrum under the condition of oil-base mud needs to be based on the difference in the pore structure of the rock, and the morphology correction should be conducted separately for different reservoir rock pore structure types.

**Figure 4:**
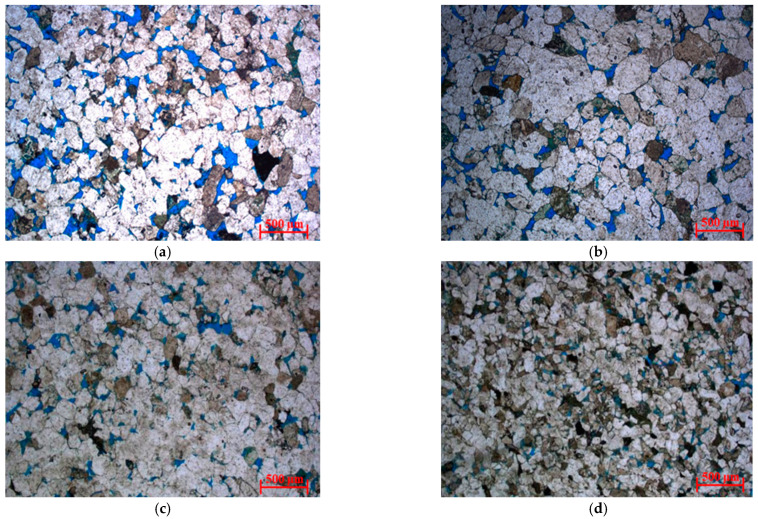
Casting sheet images of four types of rocks: (**a**) Type I (Φ = 13.9%, K = 269 mD); (**b**) Type II (Φ = 10.6%, K = 17.1 mD); (**c**) Type Ⅲ (Φ = 10.4%, K = 4.9 mD); (**d**) Type Ⅳ (Φ = 9.7%, K = 0.45 mD).

Through the observation and analysis of cast thin sections in this area, the pore structure of gas-bearing reservoir rocks can be divided into four categories according to the degree of pore development and connectivity. Figure 4a–d are the cast thin sections of typical plunger cores of reservoir rocks with four types of pore structures (hereinafter referred to as type I–IV rocks). The total porosity and permeability are the physical property test results of plunger cores. Type I rock clastic particles are well sorted, dominated by medium sand, rock pores are relatively developed, and pore connectivity is good. The clastic particles of type II rock are well sorted, dominated by medium sand, and the rock pores are relatively developed, but the connectivity is general. The clastic particles of type III rock are moderately sorted, slightly dominated by medium sand, and the rock pores are poorly developed and unevenly distributed. Type IV rock clastic particles are moderately sorted, slightly dominated by fine sand, poor rock pore development, and poor connectivity.

**Figure 5:**
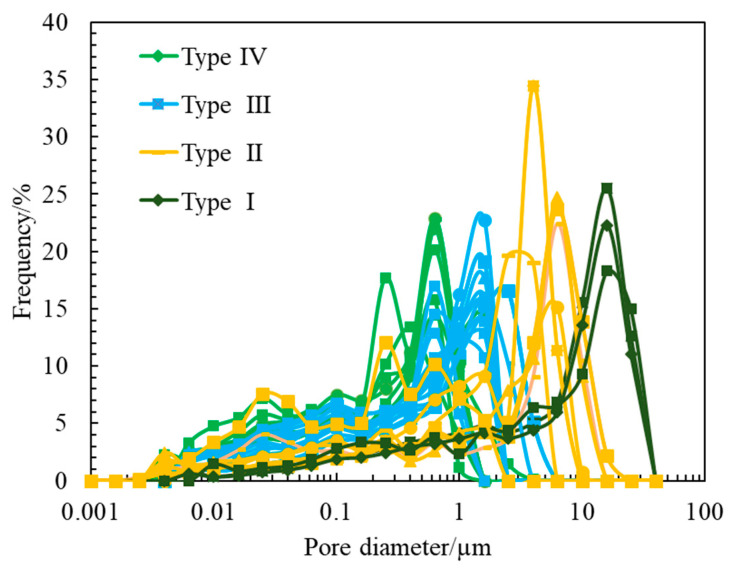
Pore-throat size distributions of four types of rocks.

Although the total porosity of cores decreases with the deterioration of pore connectivity, it is difficult to distinguish the types of reservoirs by porosity because the total porosity of cores of reservoirs with different pore structure types has little difference. Permeability reflects the seepage capacity of reservoir rocks and is a comprehensive characterization of porosity and pore structure [19]. Especially for reservoir rocks with low porosity and permeability, the permeability is primarily controlled by the pore structure in the rock, and the primary factor affecting the seepage capacity is the pores of different sizes and their matching relationship with the throat [20]. Figure 4 also shows that there are orders of magnitude differences in the permeability of reservoir cores with different pore structure types. Therefore, permeability can be used as the basis for classifying pore structure types. To facilitate the follow-up treatment, the pore structure types were divided according to the order of permeability: the permeability of type I rock is *K* > 100 mD, the permeability of type II rock is 10 mD ≤ *K* < 100 mD, the permeability of type III rock is 1 mD ≤ *K* < 10 mD, and the permeability of type IV rock is *K* < 1 mD. The results of the pore throat radius distribution calculated by the high-pressure mercury injection experiment can verify the feasibility of the pore structure classification standard. Figure 5 shows the pore throat radius distribution of 36 sandstones of four types of rocks divided according to the order of permeability, with obvious differences in pore throat dimensions. The main peak mean values of pore throat radius of type I–IV rocks are 16.13 µm, 4.74 µm, 1.35 µm and 0.55 µm, respectively. Although the main peak and mean values of the pore throat radius of a few cores are quite different, the overall pore throat radius distribution corresponds well with the four types of rocks. In addition, the pore throat radius distribution calculated by the high-pressure mercury injection experiment also explains the difference between the apparent porosity and total porosity of cast thin sections. For type IV rocks, micropores are relatively developed.

**Figure 6:**
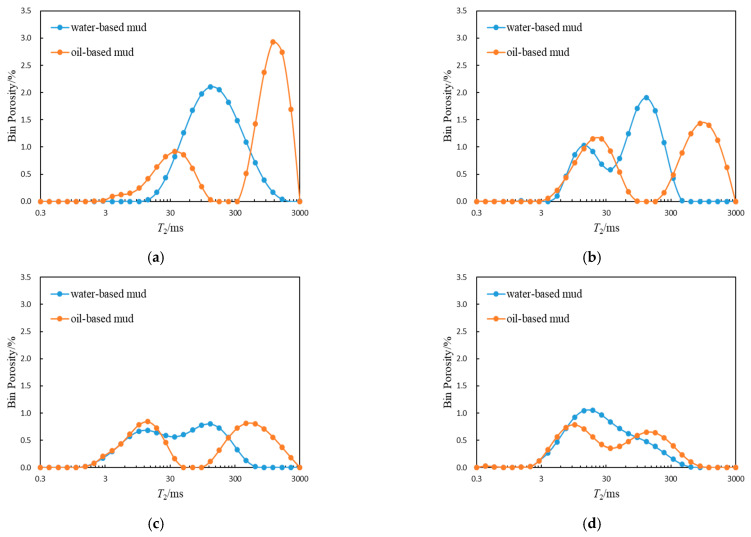
Comparisons of *T*_2_ spectrum of four types of rocks in different mud environments: (**a**) Type I; (**b**) Type II; (**c**) Type III; (**d**) Type IV.

The permeability affects the depth of oil-based mud filtrate invading the formation [21], and the pore fluid of rocks in different permeability formations has their own unique distribution characteristics [22]. Therefore, the *T*_2_ spectrum of NMR logging will be significantly different for reservoir rocks with different types of pore structures under the condition of oil-based mud. To compare the difference in *T*_2_ spectrum characteristic parameters between oil-based mud wells and water-based mud wells under similar formation physical parameters, this study selected the typical *T*_2_ spectrum of nuclear magnetic resonance logging of four types of rocks (Figure 6), and the analysis of characteristic parameters is shown in Table 1. For the *T*_2_ spectrum of water-based mud wells, the proportion of spectral area greater than 600 ms is very low. Therefore, the invasion degree of the oil-based mud filtrate can be reflected by counting the proportion of porosity with transverse relaxation times greater than 600 ms. The *T*_2_ spectrum of nuclear magnetic resonance logging of type I rocks under different mud conditions had the greatest difference, indicating that for cores with good pore connectivity, the mud filtrate had the strongest change to the original formation fluid. Under the conditions of oil-based mud and water-based mud, the difference in *T*_2_ spectrum of type II–IV rocks gradually decreased, indicating that with the deterioration of pore structure, the influence of mud filtrate on the fluid of the original formation gradually decreases, yet the shape of the *T*_2_ spectrum still needed to be corrected.

### 2.3. Determination of Optimal T_2cut-off_

In this study, the core NMR experiment of water saturation and centrifugation was conducted on 29 cores to determine the optimal *T*_2*cut-off*_ value in the study area. The specific parameters are shown in Table 2. According to the cut-off value, the *T*_2_ spectrum of NMR logging was divided into macropores and small pores. The *T*_2_ spectrum of NMR logging less than the *T*_2_ cut-off value represents the small pores and retains its original shape; the *T*_2_ spectrum of NMR logging greater than the *T*_2_ cut-off value represents the macropore part, which needs morphological correction. The size of the *T*_2*cut-off*_ value was related to the density of the core. With increasing porosity and permeability, the *T*_2*cut-off*_ value also tended to increase, yet the fitting degree was relatively low (determination coefficient R2 = 0.38). Although the *T*_2*cut-off*_ value had a good correlation with the geometric mean value of the *T*_2_ spectrum (determination coefficient R2 = 0.68), the geometric mean value of the *T*_2_ spectrum of the oil-based mud well was quite different from the geometric mean value of the *T*_2_ spectrum of saturated water, so it cannot be applied to actual logging interpretation. Therefore, the average value of the experimental *T*_2*cut-off*_ value of 29 cores of 17.48 ms was finally selected as the optimal *T*_2*cut-off*_ value.

### 2.4. Case Studies

**Figure 7:**
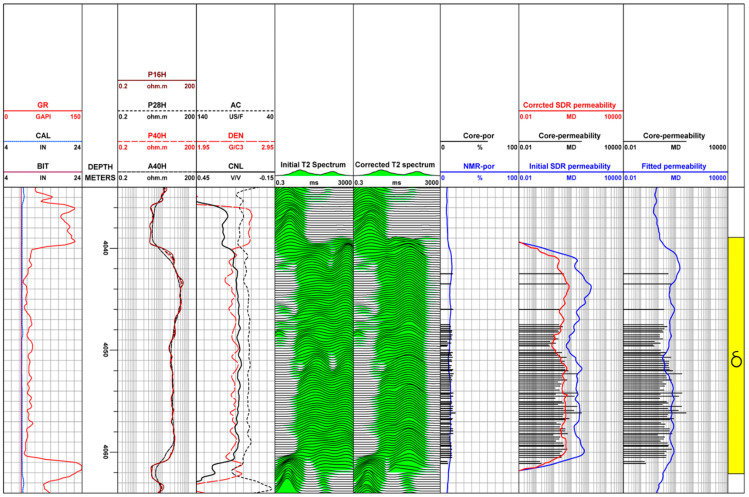
A field example of correcting the invasion of oil-based mud to field NMR logging in well B. In the first track, the displayed curves are gamma-ray (GR), borehole diameter (CAL), and bit diameter (BIT). The second track is the depth. The third is resistivity curve. The acoustic transit time log (AC), the density log (DEN), compensated neutron log (CNL) are shown in the fourth track. The fifth track is the initial measured *T*_2_ spectrum of NMR logging before correction (Initial *T*_2_ spectrum). The *T*_2_ spectrum of nuclear magnetic resonance logging (Corrected *T*_2_ spectrum) under the condition of water-based mud corrected by the method proposed in this study is displayed in the sixth track. The NMR calculated porosity (NMR-por) and core analysis porosity (Core-por) are shown in the seventh track and have good consistency. The Corrected SDR permeability is calculated permeability from the corrected *T*_2_ spectrum by using Schlumberger-doll-Research (SDR) model, and the Initial SDR permeability is estimated permeability from the Initial *T*_2_ spectrum by using the SDR model, the Core-permeability is derived permeability from core analysis. The ninth track is the permeability calculated by using the traditional porosity-permeability relationship (Fitted permeability). Good consistency of estimated permeability from the Corrected *T*_2_ spectrum with core derived permeability illustrates the reliability of the proposed method.

**Figure 8:**
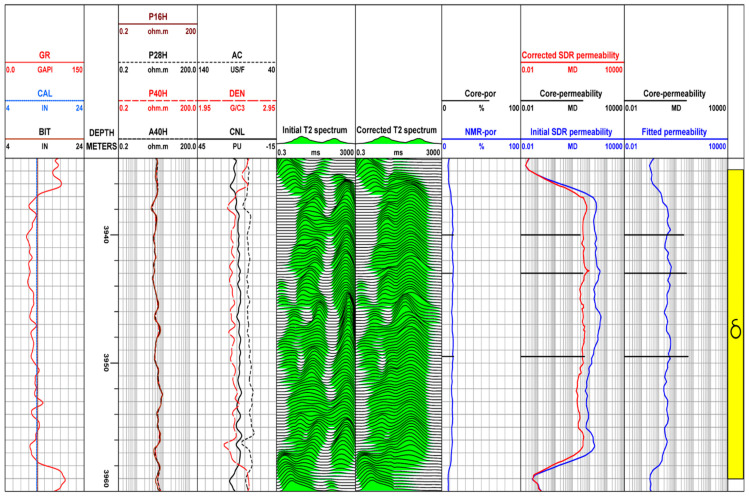
A field example of correcting the invasion of oil-based mud to field NMR logging in well C. In the first track, the displayed curves are gamma ray (GR), borehole diameter (CAL), and bit diameter (BIT). The second track is the depth. The third is resistivity curve. The acoustic transit time log (AC), the density log (DEN), compensated neutron log (CNL) are shown in the fourth track. The fifth track is the initial measured *T*_2_ spectrum of NMR logging before correction (Initial *T*_2_ spectrum). The *T*_2_ spectrum of nuclear magnetic resonance logging (Corrected *T*_2_ spectrum) under the condition of water-based mud corrected by the method proposed in this study is displayed in the sixth track. The NMR calculated porosity (NMR-por) and core analysis porosity (Core-por) are shown in the seventh track and have good consistency. The Corrected SDR permeability is calculated permeability from the corrected *T*_2_ spectrum by using Schlumberger-doll-Research (SDR) model, and the Initial SDR permeability is estimated permeability from the Initial *T*_2_ spectrum by using SDR model, the Core-permeability is derived permeability from core analysis. The ninth track is the permeability calculated by using the traditional porosity-permeability relationship (Fitted permeability). Good consistency of estimated permeability from Corrected *T*_2_ spectrum with core derived permeability illustrates the reliability of the proposed method.

According to the morphological correction method of the *T*_2_ spectrum of nuclear magnetic resonance logging under the condition of oil-based mud, the *T*_2_ spectrum data of nuclear magnetic resonance logging under the condition of oil-based mud and water-based mud extracted by the multi-dimensional matrix method were used to calibrate four types of rocks, and the coefficients and constants of multivariate linear function corresponding to each type of rock were obtained. The executable program was compiled based on the FORTRAN language to correct the *T*_2_ spectrum of NMR logging of oil-based mud wells (well B and well C) in the East China Sea. The results are shown in Figure 7 and Figure 8.

According to the core porosity and permeability results, Figure 7 shows that the pore structure type of the well B reservoir in the study area is type III, belonging to a low permeability reservoir. It can be seen from the fifth track that the measured *T*_2_ spectrum (Initial T2 spectrum) shows a continuous bimodal shape, representing that the maximum relaxation time of the second peak of the oil-based mud filtrate signal reaches 3000 ms, showing the characteristics of the macropore structure, which is seriously inconsistent with the core analysis results. Compared with the nuclear magnetic resonance *T*_2_ spectrum before correction, the macropore part of the corrected nuclear magnetic *T*_2_ spectrum (Corrected T2 spectrum) moves to the left, showing a continuous single peak shape, and the “tailing” phenomenon disappears, eliminating the fake long transverse relaxation time signal caused by the invasion of the oil-based mud filtrate. In addition, under the condition of oil-based mud, the permeability calculated by the *T*_2_ spectrum of nuclear magnetic resonance logging before correction (Initial SDR permeability) is obviously larger than that of core analysis (Core-permeability), and the permeability calculated by *T*_2_ spectrum of nuclear magnetic resonance logging after correction (Corrected SDR permeability) is smaller, which is in good agreement with the permeability results of core analysis.

According to the core analysis results, Figure 8 shows that the pore structure of well C is better than that of adjacent well B, and the pore structure is type II. It can be seen from the results of the fifth track that due to the improvement of pore structure and the increase of the number of macropores, the phenomenon of oil-based mud filtrate invading the formation pores is more serious. The nuclear magnetic *T*_2_ spectrum shows a double peak or three peak shape, and the last two peaks even produce fractures and empty white belts. Compared with the nuclear magnetic resonance *T*_2_ spectrum before correction, the macropore part of the corrected nuclear magnetic *T*_2_ spectrum moves to the left, which not only eliminates the macropore illusion caused by the invasion of oil-based mud filtrate but also eliminates the phenomenon of the blank zone in the middle, making the nuclear magnetic *T*_2_ spectrum continuous. In addition, under the condition of oil-based mud, the permeability calculated by the *T*_2_ spectrum of nuclear magnetic resonance logging after correction also decreases, which is in good agreement with the permeability results of core analysis.

**Figure 9:**
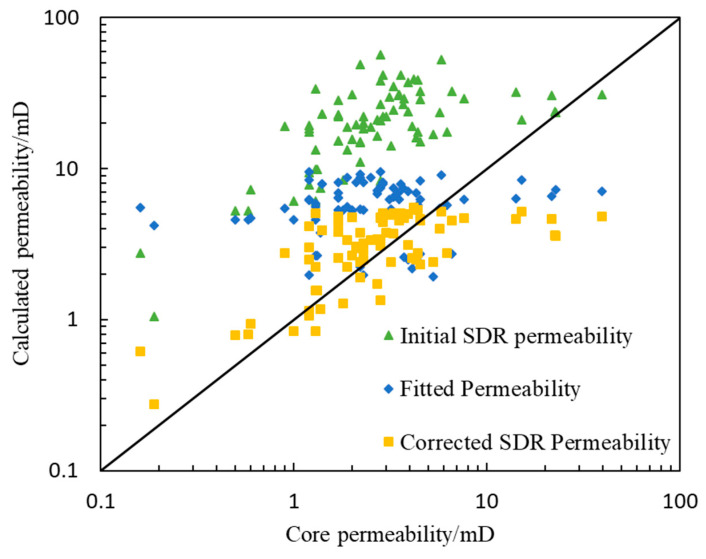
Comparison of permeability calculated by various methods and core analysis results.

To further illustrate the reliability of the *T*_2_ spectrum morphology correction results, the permeability results calculated by the *T*_2_ spectrum of NMR logging before and after correction were compared with the permeability of core samples in wells B and C (Figure 9). The permeability calculated by the uncorrected *T*_2_ spectrum of nuclear magnetic resonance logging obviously deviates from the 45° line, and the error with the core analysis result is approximately one order of magnitude (relative error 779.37%). The permeability calculated by the *T*_2_ spectrum of corrected NMR logging is more consistent with that of core samples (relative error 34.32%), which is better distributed on both sides of the 45° line. Compared with the permeability calculated by core porosity and permeability fitting (relative error 82.98%), the overall accuracy of permeability calculated by the corrected nuclear magnetic *T*_2_ spectrum improved by 48.66%. The results show that the morphological correction method of the *T*_2_ spectrum of NMR logging under the condition of oil-based mud is reliable, and the influence of oil-based mud invasion on the *T*_2_ spectrum of NMR logging has been eliminated. The corrected NMR *T*_2_ spectrum can be used as the NMR *T*_2_ spectrum measured under the condition of water-based mud, which lays a foundation for subsequent processing and interpretation.

## 3. Materials and Methods

The logging data used in this study were collected from five wells in the Xihu Sag, East China Sea basin, including three water-based mud wells and two oil-based mud wells (Table 3). The oil-based mud wells and water-based mud wells in the study area are measured by *T*_2_ spectrum with the MREx nuclear magnetic resonance logging tool developed by Baker Hughes company, and the echo space is 0.6 ms. The maximum radial detection radius of the instrument is approximately 11.43 cm, which primarily detects the hydrogen signal of the fluid in the formation invasion zone. In addition, rock cast thin section image data and 29 rocks used for NMR experiments were obtained from these wells, and the data were provided by CNOOC Shanghai Branch.

### 3.1. Workflow of T_2_ Spectrum Shape Correction Method

**Figure 10:**
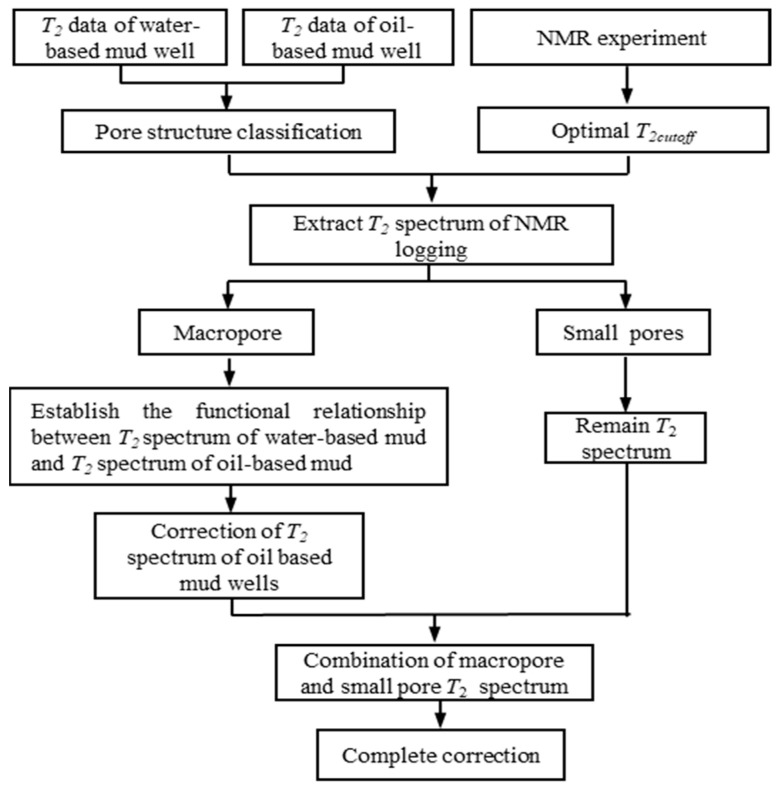
Workflow of the *T*_2_ spectrum shape correction method for NMR logging under oil-based mud conditions.

Since there are reliable technical means to evaluate reservoir parameters using nuclear magnetic resonance *T*_2_ spectrum measured under water-based mud conditions, this study establishes the correlation between the *T*_2_ spectrum of oil-based mud wells and the *T*_2_ spectrum of water-based mud wells. The *T*_2_ spectrum morphology of oil-based mud wells was corrected to the *T*_2_ spectrum morphology of water-based mud wells. The realization method is shown in Figure 10. First, according to the difference in pore structure, the reservoir rocks were divided into four types. Second, the multidimensional matrix technology was used to construct the NMR *T*_2_ spectrum sample library in the study area. Core saturation and centrifugal NMR experiments were used to determine the optimal *T*_2_ cutoff value in the study area and then divide the large and small pores. Finally, under the condition of a similar pore structure, the multivariate linear function relationship of the *T*_2_ spectrum of rock macropores in water-based mud wells and oil-based mud wells was established, and the correction of the rock *T*_2_ spectrum morphology of oil-based mud wells was realized.

### 3.2. Extraction of T_2_ Spectrum from NMR Logging by the Multidimensional Matrix Method

To correct the morphology of the *T*_2_ spectrum of formation nuclear magnetic resonance logging in an oil-based mud environment, it is necessary to determine the *T*_2_ spectrum of nuclear magnetic resonance logging in a water-based mud environment, to establish the correlation between the *T*_2_ spectrum of oil-based mud wells and the *T*_2_ spectrum characteristics of water-based mud wells. However, for a well, nuclear magnetic resonance *T*_2_ spectrum measurements in water-based mud and oil-based mud environments cannot be conducted at the same time. Additionally, due to the relatively small number of cores drilled during offshore operation, a large number of different types of mud invasion and nuclear magnetic resonance joint measurement experiments cannot be conducted. To obtain the nuclear magnetic *T*_2_ spectrum in water-based mud and oil-based mud with similar pore structures, this study proposes the use of a multi-dimensional matrix method to construct a nuclear magnetic *T*_2_ spectrum sample library in the study area to obtain *T*_2_ spectrum data of NMR logging of reservoir rocks under the conditions of water-based mud and oil-based mud with similar curve values, such as porosity, permeability, natural gamma ray (GR), acoustic transit time log (AC), compensated neutron log (CNL), and density log (DEN). For the wells with two types of drilling fluid, the formation porosity is calculated by NMR logging. For water-based mud wells, because the *T*_2_ spectrum of nuclear magnetic resonance logging can accurately calculate the permeability, the permeability calculated by the *T*_2_ spectrum is used as the permeability of water-based mud wells; for oil-based mud wells, the permeability calculated by the *T*_2_ spectrum is quite different from the core analysis results, but the difference between the permeability calculated by the porosity and permeability fitting method and the core analysis results is relatively small. Therefore, the permeability calculated by the porosity and permeability fitting method was used as the permeability of oil-based mud wells. The formula of the multidimensional matrix method is as follows:

(2) A=[M(ϕ, K, GR, AC, CNL, DEN), N(ϕ,K,GR,AC,CNL,DEN)]

where, *A* is the total data matrix, including nuclear magnetic *T*_2_ spectrum data in oil-based mud and water-based mud environments. *M* is the NMR *T*_2_ spectrum sample library in an oil-based mud environment, and *N* is the NMR *T*_2_ spectrum sample library in a water-based mud environment.

According to the different logging curve values of typical reservoirs in the study area, the nuclear magnetic *T*_2_ spectrum data under the conditions of oil-based mud and water-based mud were classified into the corresponding sample library point by point, and the *T*_2_ spectrum sample libraries under the conditions of water-based mud and oil-based mud are established. Therefore, for a certain depth point in the formation of oil-based mud wells, the *T*_2_ spectrum of similar pore structure of adjacent water-based mud wells can be obtained, and then the characteristics of *T*_2_ spectrum under different mud types can be compared to establish the correlation between them. This method avoids the problems of limited core NMR experimental data, poor representativeness, and a long experimental cycle, and is convenient for the subsequent calibration of the *T*_2_ spectrum morphology correction model.

### 3.3. Development of T_2_ Spectrum Shape Correction Model for Oil-Based Mud Wells

Both NMR *T*_2_ spectrum data and core mercury injection data can be used to analyze the pore throat size of reservoir rocks, and the two have good consistency [23]. To quantitatively analyze pores of different sizes in rocks, some researchers have proposed a method to characterize the pore size by using the porosity of the *T*_2_ spectrum interval of nuclear magnetic resonance logging [24]. Predecessors usually give seven fixed transverse relaxation time values to characterize the pore structure and pore size distribution information of reservoir rock, namely 1.0 ms, 3.0 ms, 10.0 ms, 33.0 ms, 100.0 ms, 300.0 ms, and 1000.0 ms, and the NMR logging *T*_2_ spectrum is divided into eight porosity intervals (named 8 bins) [25]. Each interval reflects different pore sizes, the short transverse relaxation time represents small pores, and the long transverse relaxation time represents large pores.

Under the actual drilling differential pressure, macropores contribute the most to the permeability of the reservoir [26]. Mud filtrate primarily invades the macropores to displace the movable fluid (including movable water and oil and gas) within the detection range, while mud filtrate invades the small pores less, and the bound fluid will not change basically. Therefore, the invasion of mud filtrate only has a great impact on the *T*_2_ spectrum of NMR logging corresponding to macropores but has little impact on the *T*_2_ spectrum of NMR logging of bound fluid in small pores [27]. Therefore, this study corrected the movable fluid part of the *T*_2_ spectrum of formation NMR logging after the invasion of oil-based mud filtrate and combined it with the *T*_2_ spectrum of NMR logging of the original bound fluid part to obtain the complete NMR *T*_2_ spectrum after morphological correction [28].

Considering that the invasion of oil-based mud had an impact on each pore component in the macropore space, and the average *T*_2*cut-off*_ value is 17.48 ms, to characterize the impact of oil-based mud filtrate on the *T*_2_ spectrum of NMR logging, this study defines only four transverse relaxation times: 33.0 ms, 100.0 ms, 300.0 ms and 1000.0 ms to divide the NMR *T*_2_ spectrum. Combining the *T*_2*cut-off*_ value and the maximum transverse relaxation time value, the *T*_2_ spectrum can be divided into five intervals ([17.48, 33.0] ms, [33.0, 100.0] ms, [100.0, 300.0] ms, [300.0, 1000.0] ms, [1000.0, 3000.0] ms), and the pore composition percentage of each interval can be calculated, as shown in Equations (3)–(5) [29]:

(3) $$X1=\frac{\int_{T_{2cutoff}}^{T_{2\left(1\right)}}S\left(T\right)dt}{\int_{T_{2min}}^{T_{2max}}S\left(T\right)dt}$$

(4) $$Xi=\frac{\int_{T_{2\left(i-1\right)}}^{T_{2\left(i\right)}}S\left(T\right)dt}{\int_{T_{2min}}^{T_{2max}}S\left(T\right)dt}\;i=2,3,4$$

(5) $$X5=\frac{\int_{T_{2\left(4\right)}}^{T_{2max}}S\left(T\right)dt}{\int_{T_{2min}}^{T_{2max}}S\left(T\right)dt}$$

where, Xi is the percentage of pore components in the nuclear magnetic *T*_2_ spectrum. The values of *T*_2*min*_ and *T*_2*max*_ are 0.3 ms and 3000 ms, respectively; *T*_2*cutoff*_ is the optimal *T_2__cut-off_* value obtained from the core NMR experiment, which is 17.48 ms in this study; *T*_2*(i)*_ are the four *T*_2_ relaxation time values defined above (33.0 ms, 100.0 ms, 300.0 ms and 1000.0 ms respectively); and *S(T)* are the pore distribution functions of the nuclear magnetic *T*_2_ spectrum.

Equations (3)–(5) can be used to calculate the percentages of five pore components according to the *T*_2_ spectrum of NMR logging after the invasion of oil-based mud filtrate. The amplitude corresponding to each relaxation time of the measured NMR *T*_2_ spectrum under the condition of water-based mud with *T*_2_ relaxation time greater than 17.48 ms was defined as the dependent variable, and the percentage of five pore components was used as the independent variable. Therefore, the multivariate linear function relationship between the amplitude of each point of *T*_2_ spectrum composition of NMR logging under the condition of water-based mud and the five pore components of *T*_2_ spectrum of NMR logging under the condition of oil-based mud was established. Using this functional relationship, the amplitude of the nuclear magnetic resonance *T*_2_ spectrum under water-based mud conditions with different relaxation times can be calculated from the nuclear magnetic resonance logging *T*_2_ spectrum under oil-based mud conditions. The function relationship is shown in Equation (6):

(6) $$A_{1}=a_{11}X_{1}+a_{12}X_{2}+a_{13}X_{3}+a_{14}X_{4}+a_{15}X_{5}+b_{1}\;A_{2}=a_{21}X_{1}+a_{22}X_{2}+a_{23}X_{3}+a_{24}X_{4}+a_{25}X_{5}+b_{2}\;A_{3}=a_{31}X_{1}+a_{32}X_{2}+a_{33}X_{3}+a_{34}X_{4}+a_{35}X_{5}+b_{3}\;\ldots \;A_{i}=a_{i1}X_{1}+a_{i2}X_{2}+a_{i3}X_{3}+a_{i4}X_{4}+a_{i5}X_{5}+b_{i}$$

where, *A_i_* represents the amplitude value corresponding to the *i* th time distribution point of the *T*_2_ spectrum of NMR logging after correction, and the value of *i* is the number of distribution points of the *T*_2_ spectrum of NMR logging; $X_{1},X_{2}\ldots \ldots .\;X_{15}$ is the percentage component of five pore components divided according to the corresponding reservoir type under the condition of oil-based mud; $a_{1},a_{2}\ldots \ldots .\;a_{15}$ is the coefficient corresponding to the multivariate linear function corresponding to the *i* th distribution point, and its value is calibrated by *T*_2_ spectrum data of nuclear magnetic resonance logging of water-based mud and oil-based mud in the sample library; $b_{1},b_{2}\ldots \ldots .\;b_{15}$ is the constant corresponding to the multivariate linear function corresponding to the *i* th distribution point, and its value (Supplementary Materials, Tables S1–S4)is calibrated by *T*_2_ spectrum data of nuclear magnetic resonance logging of water-based mud and oil-based mud in the sample library.

## 4. Conclusions

In the process of drilling, the invasion of oil-based mud filtrate has a serious impact on the *T*_2_ spectrum of NMR logging, so NMR logging data cannot be directly used to evaluate reservoir parameters. Because the invasion degree of oil-based mud filtrate to reservoirs with different pore structures is different, through the analysis of cast thin sections, physical property experiments, and high-pressure mercury injection experiments, it is proposed to divide the reservoir rocks into four types based on the permeability range.

Based on the difference between the *T*_2_ spectrum of nuclear magnetic resonance logging in water-based mud wells and oil-based mud wells, the corresponding morphological correction model of the nuclear magnetic resonance *T*_2_ spectrum was established. By comparing the permeability, pore fitting permeability and core analysis permeability calculated by *T*_2_ spectrum measured by NMR before and after correction, it was found that the permeability calculated after *T*_2_ spectrum morphology correction is the most accurate, which improved the accuracy of calculating permeability by using NMR logging data in oil-based mud wells, it also laid a foundation for further NMR logging of data to evaluate other reservoir parameters.

The proposed correction model has been successfully applied in sandstone reservoirs, which lays a solid foundation for the morphological correction of the *T*_2_ spectrum of NMR logging under the condition of oil-based mud in shale oil reservoirs. Different from sandstone reservoirs, when applying the model proposed in this study to NMR *T*_2_ spectrum correction of shale oil reservoirs, it is necessary to comprehensively consider the complex pore network composition of shale oil reservoirs and the response mechanism of NMR logging under complex fluid occurrence modes.

## Figures and Tables

**Table 1:** *T*_2_ spectrum characteristic parameters of four types of rocks in different mud environments.

Rock Structure Type	*T*_2_ Peak/ms	*T*_2_ Geometric Average/ms	Proportion of Pores Greater than 600 ms/%
I (Water-based mud)	125.26	140.97	8.07
I (Oil-based mud)	1156.99	363.55	66.12
II (Water-based mud)	125.26	58.92	0
II (Oil-based mud)	842.16	148.35	42.61
III (Water-based mud)	125.26	36.91	0.17
III (Oil-based mud)	446.21	82.47	26.85
IV (Water-based mud)	13.56	25.98	0.11
IV (Oil-based mud)	125.26	35.17	1.48

**Table 2:** Statistical table of physical property parameters of 29 sandstone core samples.

Core No	*T*_2cutoff_/ms	Permeability/mD	NMR Porosity/%	*T*_2_ Geometric Average/ms	Irreducible Water Saturation/%
1	10.35	10.80	17.09	11.91	38.74
2	15.70	15.30	16.73	20.96	34.25
3	13.66	17.51	16.57	16.06	44.48
4	44.48	522.3	21.10	101.65	23.12
5	23.81	63.10	18.4	35.50	31.74
6	12.75	5.40	12.35	6.63	55.37
7	22.21	18.45	17.26	22.97	55.37
8	12.75	3.20	12.88	9.48	53.47
9	5.54	9.90	14.9	8.50	43.86
10	15.70	4.20	14.38	12.39	51.74
11	18.04	4.20	8.78	2.36	77.81
12	14.64	4.83	15.11	8.32	57.03
13	12.75	2.18	14.22	9.98	54.19
14	22.21	0.43	9.82	16.38	44.48
15	19.33	0.20	8.66	5.57	73.46
16	5.54	6.56	14.54	5.51	53.65
17	12.75	1.73	13.39	10.08	51.82
18	16.83	1.23	12.13	9.91	55.3
19	38.72	68.40	17.58	37.19	46.32
20	18.04	2.87	15.47	14.73	49.42
21	4.82	1.65	13.32	6.40	47.75
22	5.94	39.40	14.11	13.88	38.10
23	11.09	2.50	13.65	9.40	51.29
24	10.35	18.20	15.64	15.37	42.69
25	6.36	3.25	13.3	8.97	44.96
26	3.65	2.85	11.09	6.33	43.44
27	27.36	29.70	18.31	29.38	39.56
28	47.68	1246.0	22.60	158.28	18.58
29	33.70	145.9	18.51	69.84	26.81

**Table 3:** Summary of the wells logging information.

Well.	Mud Type	Lithology	Wellbore Orientation	Logs
A	WBM	Sand	Vertical	WL 3combo ^1^+WLNMR
B	OBM	Sand	Vertical	LWD 3combo+WLNMR
C	OBM	Sand	Vertical	LWD 3combo+WLNMR
D	WBM	Sand	Vertical	WL 3combo+WLNMR
E	WBM	Sand	Vertical	WL 3combo+WLNMR

^1^ 3 combo includes gamma-ray, density-neutron, resistivity.

## Data Availability

Data available on request to the corresponding author.

## Supplementary Materials

The following are available online. Tables S1–S4: The coefficients and constant matrix values used in the four types of rocks in the calibration process.

## Sample Availability

Samples of the compounds are available from the authors.
