# Supplementary material for: Study on Nuclear Magnetic Resonance Logging T2 Spectrum Shape Correction of Sandstone Reservoirs in Oil-Based Mud Wells
Source: Molecules. 2021 Oct 8;26(19):6082. doi: 10.3390/molecules26196082 (PMC8512091; doi:10.3390/molecules26196082)
Supplement: Supplementary file 1 [file molecules-26-06082-s001.zip › molecules-1383038-supplementary.pdf]

Supplementary Materials

Study on Nuclear Magnetic Resonance Logging T_2 Spectrum Shape Correction of Sandstone Reservoirs in Oil-Based Mud Wells

Jianmeng Sun [1,2], Jun Cai [3], Ping Feng [2,*], Fujing Sun [2], Jun Li [1], Jing Lu [1] and Weichao Yan [2]

1 State Energy Center for Shale Oil Research and Development., Beijing 100083, China; sunjm@upc.edu.cn (J.S.), lujing.syky@sinopec.com (J.L.), lijun67.syky@sinopec.com (J.L.),
2 School of Geosciences, China University of Petroleum (East China), Qingdao 266580, China; sunjm@upc.edu.cn (J.S.), b20010072@upc.edu.cn (F.S.), yanweichaoqz@163.com (W.Y.)
3 Shanghai Branch of CNOOC Ltd., Shanghai 200030, China; caijun@cnooc.com.cn
* Correspondence: upcfengping@163.com; Tel.: +86-17863960551

Table S1. The value of the coefficient and constant matrix used in the first type of rock in the calibration process.

ai1	ai2	ai3	ai4	ai5	bi
0.365752347	0.858019712	0.229512726	-0.460149893	-0.1841621	-0.176871749
0.598988833	0.70840873	0.747297003	0.133509884	-0.632480086	0.499190336
0.838000525	0.205955701	1.208608981	1.030337093	-1.091720355	1.34206528
1.047085388	-0.427302763	1.327616727	1.737164207	-1.310274278	1.88579423
1.195704473	-0.932418078	1.003644873	1.923045067	-1.183229711	1.865350647
1.260158663	-1.141404299	0.36351085	1.589611499	-0.804850318	1.343354934
1.230596487	-1.025009002	-0.332870094	0.965429012	-0.360331652	0.593650954
1.117536554	-0.674399523	-0.835768881	0.320070135	-0.008267836	-0.08814093
0.95355296	-0.236850109	-1.009644881	-0.175421938	0.18196112	-0.523951831
0.78368456	0.133792074	-0.871278734	-0.452265296	0.197268835	-0.673451464
0.567719065	0.407217492	-0.567708534	-0.350440794	0.082492446	-0.494642584
0.395625278	0.573661248	-0.31215732	-0.170705683	-0.040900636	-0.287373489
0.29079817	0.584507995	-0.120692484	-0.05776005	-0.10965797	-0.145802839
0.220905419	0.457347022	0.00749889	0.015857703	-0.127625848	-0.046324037
0.15039618	0.300110668	0.074570829	0.06074228	-0.11168876	0.022219195
0.07837835	0.136358287	0.060897744	0.056540727	-0.066502532	0.036591808
0.006183281	-0.028771969	-0.017730498	0.009630838	-0.002415313	0.001949996

Table S2. The value of the coefficient and constant matrix used in the second type of rock in the calibration process.

ai1	ai2	ai3	ai4	ai5	bi
0.732733057	-0.308415271	-0.562361701	-0.439306724	-0.305373059	-0.522524694

0.828359794	-0.278931109	-0.689936033	-0.168413809	-0.10377057	-0.61146651
0.902233561	-0.30532734	-0.681862616	0.33661311	0.246771659	-0.568592161
0.997650126	-0.453029403	-0.532194658	0.839711891	0.542813274	-0.426203352
1.129675347	-0.655129677	-0.264650177	1.073901323	0.597120427	-0.249403818
1.264192136	-0.707153404	0.039340959	0.912655641	0.32326474	-0.081785476
1.333837149	-0.4769639	0.308549668	0.405288788	-0.164845429	0.023404922
1.281394908	-0.014779373	0.470979075	-0.214663946	-0.651561702	0.050278634
1.09378232	0.448592463	0.472710623	-0.640001265	-0.930492025	0.025743904
0.810308772	0.647182047	0.31378466	-0.675194647	-0.900687962	-0.000220232
0.502331388	0.478895639	0.136550804	-0.512288908	-0.598608024	-0.00845916
0.260940919	0.086096601	0.004338431	-0.319646427	-0.256871226	0.00303875
0.074327173	-0.129307844	-0.030534874	-0.085025852	0.012711052	0.105820445
-0.038298226	-0.099155406	0.008631921	0.09176313	0.141612906	0.215527968
-0.071198275	0.012876782	0.057838958	0.127419155	0.17070866	0.205491446
-0.05021581	0.041959142	0.057941864	0.0814442	0.113422014	0.120976933
-0.002389307	0.020466674	0.038832504	-0.006891351	0.00265519	-0.009771921

Table S3. The value of the coefficient and constant matrix used in the third type of rock in the calibration process.

ai1	ai2	ai3	ai4	ai5	bi
0.538048146	1.220847825	-0.363662739	0.357904722	0.070452603	-0.512262961
0.620606386	0.820909322	-0.165371292	0.136170798	0.21089004	-0.530971577
0.700597386	0.298198043	0.079991733	-0.051379413	0.381192447	-0.497945127
0.757881655	-0.158463133	0.310505619	-0.120652126	0.547307661	-0.356877069
0.759021661	-0.499692616	0.529912929	-0.022734719	0.710936337	-0.014155543
0.713179493	-0.654869286	0.658471291	0.171336085	0.794518594	0.393831086
0.629647607	-0.67601986	0.668690627	0.348640871	0.771720599	0.726501065
0.524099608	-0.657051822	0.575292971	0.411834955	0.653597041	0.872564735
0.414345537	-0.666397502	0.425756471	0.326920408	0.489469458	0.788898073
0.314156967	-0.748638702	0.287939592	0.131437167	0.366728079	0.521223539
0.196088916	-0.712011031	0.212181302	0.006844433	0.323048994	0.236632148
0.140994103	-0.622963918	0.106984716	-0.076521121	0.216411515	-0.016731354
0.129565371	-0.392849974	-0.025511549	-0.131320071	0.052248034	-0.170601259
0.14102767	-0.171978612	-0.135946769	-0.168064351	-0.118219433	-0.223202062
0.130973189	-0.049957972	-0.159289035	-0.144412714	-0.192471423	-0.195049904
0.078566374	0.040946699	-0.120001026	-0.048201625	-0.153474684	-0.106199889
-0.006780582	0.095210475	-0.024914475	0.072282708	-0.026695097	0.026770292

Table S4. The value of the coefficient and constant matrix used in the fourth type of rock in the calibration process.

ai1	ai2	ai3	ai4	ai5	bi

0.445472406	0.115348867	0.312876307	0.298375076	0.461635184	1.373136171
0.447366732	0.253111398	0.276543012	0.546833767	0.516466185	1.218225784
0.415640046	0.428887161	0.25496218	0.828208999	0.549649994	1.102356837
0.344230461	0.583551258	0.278282407	1.14783261	0.571914642	1.167497689
0.256475545	0.581258252	0.336797714	1.441385085	0.615156127	1.378214339
0.183562831	0.306672884	0.488979121	1.515117841	0.724570148	1.555483979
0.135759046	-0.04962297	0.617852148	1.35129623	0.762860786	1.661939717
0.108620537	-0.28023677	0.599191799	1.045409563	0.688063405	1.591781143
0.096151316	-0.303560322	0.428464734	0.691952537	0.499153568	1.28929903
0.086455658	-0.221767819	0.222762612	0.389982067	0.232394805	0.866585608
0.076217534	-0.180463224	0.095123174	0.15304516	0.042960974	0.421799014
0.070538847	-0.151337942	0.039959101	-0.023370042	-0.062323576	0.061512823
0.083175598	-0.157108991	-0.010413104	-0.119734253	-0.077216844	-0.186473867
0.101412696	-0.191404598	-0.037768275	-0.178938184	-0.03465123	-0.323113776
0.097109037	-0.162233272	-0.036572532	-0.193481543	-0.015655505	-0.31935693
0.063787346	-0.078827545	-0.011671852	-0.143184062	0.008097792	-0.221217406
-0.005017022	0.077338286	0.031840168	-0.010114646	0.032724194	-0.036100768
